# Information filtering based on corrected redundancy-eliminating mass diffusion

**DOI:** 10.1371/journal.pone.0181402

**Published:** 2017-07-27

**Authors:** Xuzhen Zhu, Yujie Yang, Guilin Chen, Matus Medo, Hui Tian, Shi-Min Cai

**Affiliations:** 1 State Key Laboratory of Networking and Switching Technology, Beijing University of Posts and Telecommunications, Beijing, 100876, China; 2 Department of Physics, University of Fribourg, Chemin du Musée 3, CH-1700 Fribourg, Switzerland; 3 Web Sciences Center, School of Computer Science and Engineering, University of Electronic Science and Technology of China, Chengdu, 611731, P.R.China; 4 Big Data Research Center, University of Electronic Science and Technology of China, Chengdu, 611731, P.R.China; Shanghai University of Finance and Economics, CHINA

## Abstract

Methods used in information filtering and recommendation often rely on quantifying the similarity between objects or users. The used similarity metrics often suffer from similarity redundancies arising from correlations between objects’ attributes. Based on an unweighted undirected object-user bipartite network, we propose a Corrected Redundancy-Eliminating similarity index (CRE) which is based on a spreading process on the network. Extensive experiments on three benchmark data sets—Movilens, Netflix and Amazon—show that when used in recommendation, the CRE yields significant improvements in terms of recommendation accuracy and diversity. A detailed analysis is presented to unveil the origins of the observed differences between the CRE and mainstream similarity indices.

## Introduction

Not so long time ago, people had to arduously travel around many stores to search for what they needed. Limited by the travel distance, number of available stores, and search costs in general, one often had to accept choices that did not meet the expectations satisfactorily. Information technologies such as the Internet [1, 2], World Wide Web [3, 4] and smart mobile devices [5, 6] have revolutionized the shopping behavior with most of the conceivable goods just a few clicks away. However, these unlimited possibilities have exposed the customers to yet another problem: that of information overload. The limited information processing capability of individuals made an additional layer of online shopping experience necessary where every customer is provided with personalized recommendation [7]. The task of personalized recommendation is to find potentially suitable items for individual customers. The recommendations are typically computed based on past purchases of all customers, features of the available items, customer personal information, or often a combination of these various approaches. Nowadays, a recommendation engine is present in most successful e-commerce web sites. For example, Amazon uses customers’ purchase records to recommend books [8], Twitter uses users’ past actions to recommend who to follow [9], AdaptiveInfo uses users’ reading histories to recommend news [10], and TiVo uses users’ viewing patterns and provided ratings to recommend TV shows and movies [11].

Due to the outstanding significance of recommendation to the economy and society, significant attention has been devoted to studying its scientific basis and engineering applications (see the review articles [12–14] and the references therein). The various approaches to recommendation have been suggested, such as content-based analysis [15, 16], context-aware analysis [17], time-aware analysis [18], tag-aware analysis [19], social recommendation analysis [20], constraint-based analysis [21], spectral analysis [22], iterative refinement [23], principle component analysis [24], information core analysis [25], and hybrid methods [26]. Furthermore, collaborative filtering (CF) recommendation algorithms become highly popular due to their simplicity and effectiveness [27]. The class of algorithms based on network-based inference (NBI) [28–34] and heat conduction [35–37] becomes popular due to their flexibility and extendability.

In an unweighted undirected object-user bipartite network, two objects are thought to be similar if they are simultaneously selected by a user. The more users co-select the two objects, the more similar the objects are believed to be. The same is true for objects who are thought to be similar if they co-selected by one or more users. However, owing to the sparsity and heterogeneity of many real-world bipartite networks, similarities among pairs of objects or users are overestimated or underestimated outstandingly, which in turn impairs accuracy of the produced recommendations. In addition, overestimating object similarity arising from object attributes leads to substantial redundancy which then directly weakens the diversity and personality of the produced recommendations. To further improve the performance of recommendation methods, these problems must be comprehensively addressed. We propose a novel similarity index, which we refer to as Corrected Redundancy-Eliminating similarity index (shortly CRE), in order to improve the accuracy and diversity of recommendations. Similarly to the corrected similarity index (shortly CSI), the CRE takes into account the symmetrical nature of the underlying mass diffusion process on the bipartite network. Most importantly, CRE eliminates unexpected original and secondary similarity redundancy—a problem which is ignored by the CSI. We show that the CRE indeed improves the recommendation performance as measured by a number of standard information filtering evaluation metrics.

## Methods

### Apparent similarity problem

**Fig 1:**
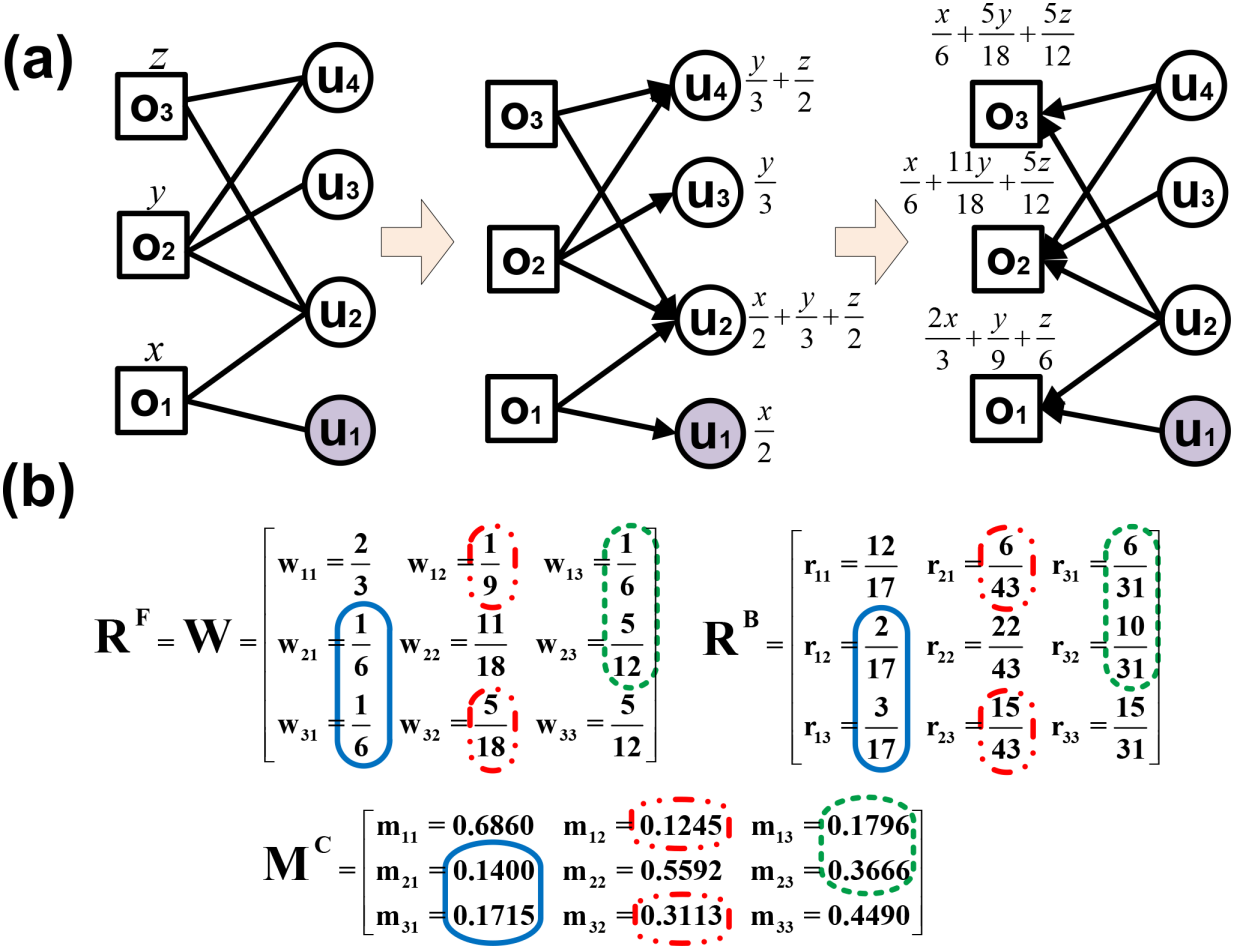
Illustrating the apparent similarity problem. (a) the description of a unweighted undirected object-user bipartite network, with objects denoted by squares and users by round circles. (b) Matrices *W*, *R*^*F*^, *R*^*B*^ and *M*^*C*^ indicate similarity matrix of NBI, forward and backward similarity proportion matrices and corrected similarity matrix, respectively. Color circles highlight the corresponding relations of similarity elements in different similarity matrices. Here element *m*_*ij*_ in *M*^*C*^ equals to $\sqrt{w_{ij}\times r_{ji}}$, with *w*_*ij*_ in *W* = *R*^*F*^ and *r*_*ji*_ in *R*^*B*^.

In traditional works based on bipartite networks (e.g. NBI), researchers naturally suppose two objects are more similar if they are commonly selected by more users. However, owing to structural sparsity and heterogeneity in bipartite networks, the apparent similarity estimations including overestimation and underestimation can happen unexpectedly. For concretely explaining the origin of such problem, we simply exemplify it in Fig 1, which has been mentioned in [33].

Concretely, in the example bipartite network shown in Fig 1(a), objects {*o*_1_, *o*_2_} and {*o*_1_, *o*_3_} are only selected by user *u*_2_ at the same time. So, the similarity from *o*_1_ to *o*_2_ is expected to the same as the one from *o*_1_ to *o*_3_, such like *w*_21_ = *w*_31_ for NBI (see *R*^*F*^ of Fig 1(b)). Nevertheless, it deviates from this expectation: the statistical sums of (mass) similarities between each object and others are assumed to be set as 1. In total three users selecting *o*_2_, only one also selects *o*_1_ and for *o*_3_ it is one in two. Accordingly, for *o*_2_, the most likely similarity only accounts for $\frac{1}{3}$ of the original, and for *o*_3_ it accounts for $\frac{1}{2}$. We inverse the similarity matrix *W* obtained from NBI, and scale it in each column (see *R*^*B*^ of Fig 1(b)). It can be found that the original (mass) similarity is overestimated between *o*_1_ and *o*_2_ (*w*_21_ > *r*_12_) or underestimated between *o*_1_ and *o*_3_ (*w*_31_ < *r*_13_). It suggests the heterogenous objects’ degrees affect the similarity estimation based asymmetrical mass diffusion of NBI. Obviously, we can solve this apparent similarity problem to introduce the symmetrical mass diffusion like CSI (more details of CSI in Sec. 2.3).

### Similarity redundancy problem

**Fig 2:**
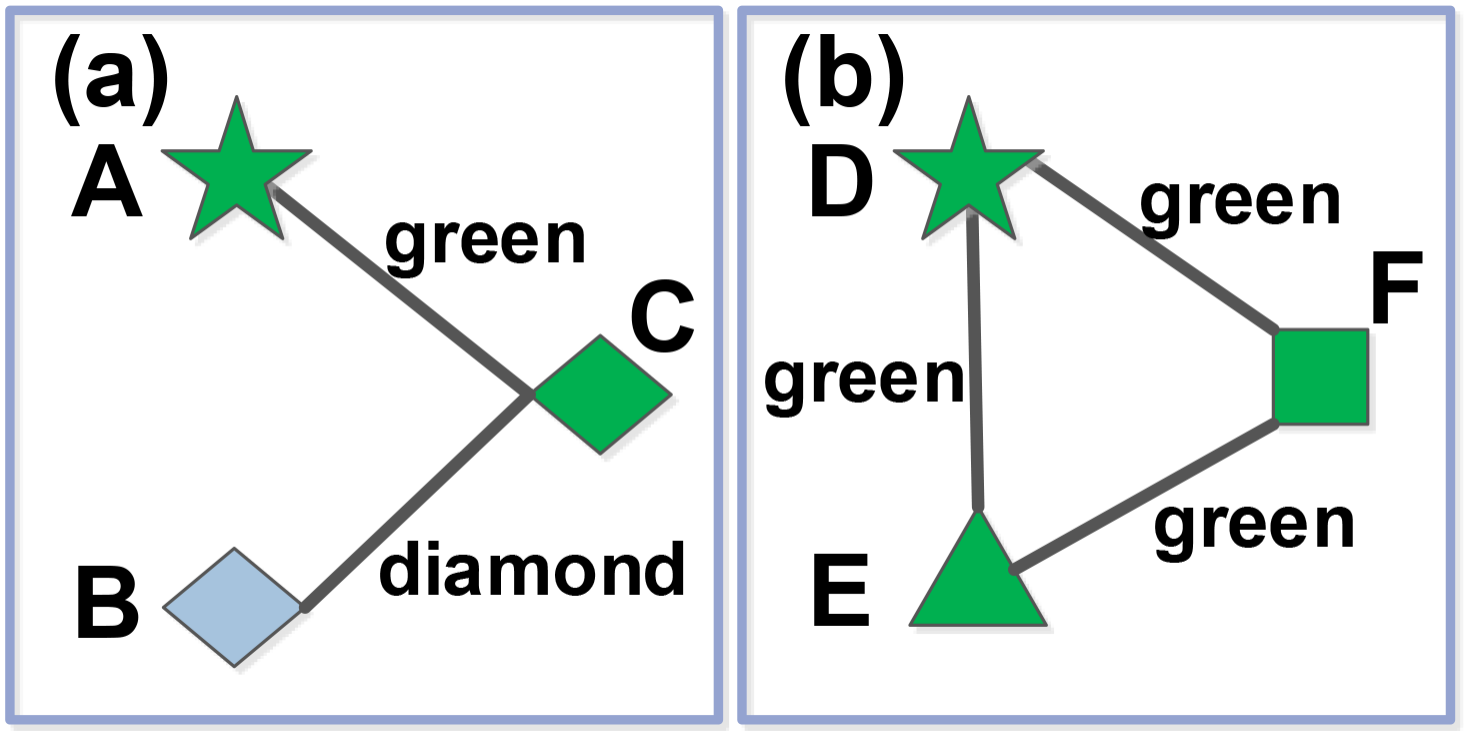
Illustration of similarity redundancy. A, B, D and E are collected objects, and C and F are the uncollected. Object pairs (A, C) and (B, C) respectively have similarities of color green and geometry diamond. So there is no similarity redundancy between A and B. However, (D, F) and (E, F) both have the similarity of color green so that D and E possess the similarity redundancy of color green.

Since CSI takes NBI as foundation, there still exists another similarity redundancy problem [31]. Basically speaking, similarity between two objects is originated from correlation between objects’ attributes. In other words, some similarities may be derived from objects’ diverse attributes and others may be deduced from objects’ same attribute, which brings in similarity redundancy and eventually harms recommendation performance, especially for diversity and personalization. Let us take Fig 2 for an example to clarify the idea.

In Fig 2, A, B, D, E represent the collected objects and C, F represent the uncollected objects, respectively. All five links, representing correlations between two objects in the object -object network, should have more or less the same weight because each of them is derived from one common attribute as labeled. Here, we may as well set the weight of each link as one unit.

Since *C* and *F* respectively has two similar collected objects, both of them are ranked with two scores based on similarity measure. However, the collected objects for *C* has two absolutely distinct attributes ‘color = green’ and ‘geometry = diamond’, while for *F* the collected objects oppose a common attribute ‘color = green’. Obviously, the two scores for *C* and *F* implies different extent of similarity. Such phenomenon is called the similarity redundancy existing ubiquitously in real recommendation systems, which recommends users many repetitions and definitely depresses diversity, personality and accuracy.

### Corrected redundancy-eliminating similarity index

The reason resulting in the apparent similarity problem is asymmetrical mass diffusion on the spares and heterogeneous BN. Much more practically, two objects are believed to be similar only if the forward similarity proportion is coherent with the backward similarity proportion. The more coherent and symmetrical, the more similar they are. Like CSI, the element *m*_*ij*_ of corrected similarity matrix *M*^*C*^ can be defined as,

$$\begin{array}{c} m_{ij}=\sqrt{r_{ij}^{F}\times r_{ji}^{B}}, \end{array}$$ (1)

where $r_{ij}^{F}$ and $r_{ji}^{B}$ are the elements of *R*^*F*^ and *R*^*B*^. The computation of $r_{ij}^{F}$ and $r_{ji}^{B}$ is according to CSI. As shown in Fig 1(b), the original apparent similarity estimations of *w*_21_ and *w*_31_ in blue solid circle. Through the corrections via *r*_12_, *r*_13_ and definition of $r_{ji}^{B}$, they are corrected as *m*_21_ and *m*_31_, respectively. Between them, the clear difference is embodied and confirms our formally expectation. Meanwhile, other similarity weight *w*_*ij*_ are transformed into *m*_*ij*_ with the same circle marker to keep the existing distinguishability, such as *w*_13_ and *w*_23_ into *m*_13_ and *m*_23_ surrounded by green dash circles.

Apart from the above apparent similarity, the redundancy similarity intrinsically originates from the common attributes among collected objects, which tightly connects them just like the *D-E* correlation (‘color = green’) in Fig 2(b). With the close correlation like *D-E*, the collected object must have strong second order correlations with the uncollected one, such as the second order correlation *D-E-F*. It causes the redundancy similarity for uncollected objects. On the contrary, if the two collected objects have weak relation, the second order correlation can be neglected, just like the *A-B-C* in Fig 2(a). Beside of the above mentioned similarity redundancy, the superposed bidirectional similarities arising from the symmetrical mass diffusion bring in secondary similarity redundancy.

Thus, after correcting apparent similarity, we should eliminate these similarity redundancies with the following definition,

**Definition 1** *With corrected similarity matrix M^C^ and tunable parameter α*, *the corrected redundancy-eliminating similarity matrix S^CRE^ is defined as follows*:

$$\begin{array}{c} S^{CRE}=M^{C}+\alpha {\left[M^{C}\right]}^{2}, \end{array}$$ (2)

*where the tunable parameter α is always negative and adjusts for different redundancy situations in diverse biparite networks*, *and* [*M*^*C*^]^2^ *represents the secondary moment of M^C^*.

If a user has selections denoted by vector *f*, the recommendations *f*′ according to corrected similarity matrix *S*^*CRE*^ can be obtained from the equation *f*′ = *S*^*CRE*^ *f*.

## Results & analysis

### Experimental results

**Fig 3:**
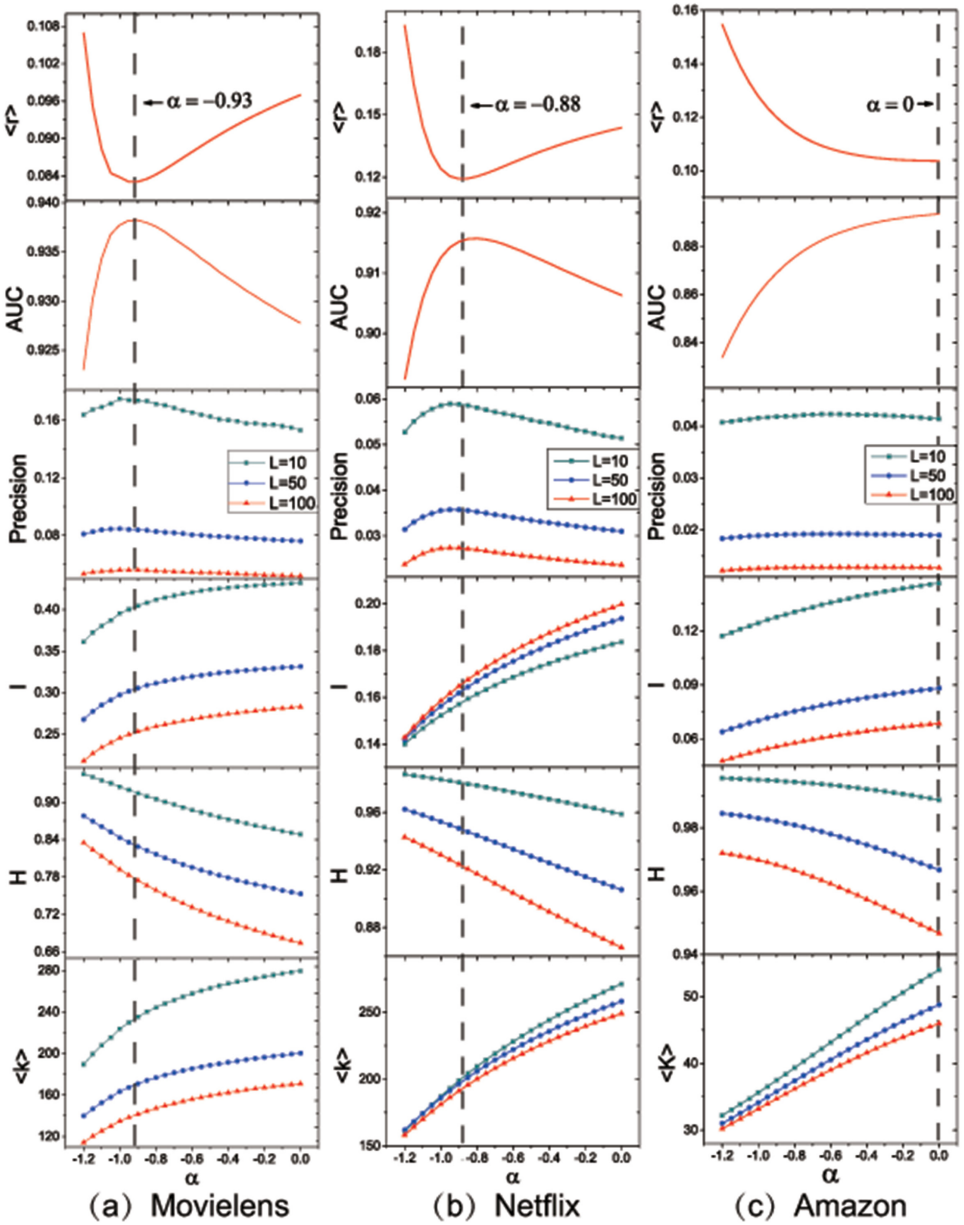
Demonstration of performances of CRE under optimal parameter (*α* = -0.93 in Movielens, -0.88 in Netflix and 0 in Amazon, from the left to the right) of lowest ranking score, with L = 10, 50 and 100. From the top to the bottom, the subgraphs exhibit ranking score, AUC, precision, intra-similarity, hamming distance and average degree in the parameter range [-1.2, 0], to confirm the performance promotion under depressing similarity redundancy, regardless of the recommendation list’s length L.

The experimental results on three benchmark datasets are averaged over ten independent random divisions. The goal of our experiment is to investigate diversity and personality under the condition of the optimal accuracy. Thus, we choose the optimal parameter when the ranking score is the lowest in each dataset, and compute six metrics (ranking score, AUC, precision, intra-similarity, hamming distance and average degree) under such parameter. In Fig 3, we plot the curves of six metrics, from the top to the bottom, with *α* in [-1.2, 0] and recommendation list’s length L = 10, 50, 100. In the same pattern, metrics curves of three datasets (Movielens, Netflix and Amazon) from the left to the right are provided. Accordingly, we first show the all evaluation metrics of six performance indices in restriction to the optimal *α* = -0.93, -0.88 and 0. These results clearly suggest that the optimal *α* definitely exists in [-1, 0], and with this restriction the other evaluation metrics (especially precision and AUC) also behave better.

Although we cannot leverage the best values of evaluation metrics with an identical parameter, the comparatively better values in restriction to higher accuracy outperform those of benchmark methods. As shown in Tables 1 and 2, the optimal parameters are subject to the lowest ranking score(〈*r*〉) for all methods (mainly HNBI, REBNI, CRE). The other evaluation metrics of precision(*P*), AUC, intrasimilarity(*I*), hamming distance(*H*), and popularity(〈*k*〉), are obtained at their optimal parameters, respectively. According to the optimal evaluation metrics, we can clearly find that the best ones emphasized in boldface are almost obtained through CRE. Distinctively, CRE perfectly acquires the most outstanding diversity and personality (see values in *I*, *H*, 〈*k*〉) with *L* = 50, 100 and also achieves much more remarkable accuracy (see values in 〈*r*〉, *P*, *AUC*) in the most cases.

**Table 1:** Performance comparison table. The optimal *α*’s of ranking score 〈*r*〉 for HNBI, RENBI, CRE are (-0.86, -0.76, -0.93) in Movielens, (-1, -0.81, -0.88) in Netflix and (-0.08, -0.53, 0) in Amazon, respectively. And the other evaluation metrics—*P* for precision, AUC, *I* for intra-similarity, *H* for hamming distance, 〈*k*〉 for popularity— take the values corresponding to the optimal *α* of 〈*r*〉. The recommendation list *L* = 50, and the sampling number *n* in *AUC* is one million. All the values are obtained by averaging over ten independent runs with different data set divisions and numbers in brackets stand for the standard deviations.

Movielens	〈*r*〉	*P*	*AUC*	*I*	*H*	〈*k*〉
CF	0.1225(0.0020)	0.0638(0.0011)	0.8990(0.0020)	0.3758(0.0008)	0.5796(0.0016)	242(0.3724)
NBI	0.1142(0.0018)	0.0670(0.0011)	0.9093(0.0016)	0.3554(0.0008)	0.6185(0.0013)	234(0.3925)
HNBI	0.1075(0.0018)	0.0693(0.0012)	0.9144(0.0014)	0.3392(0.0010)	0.6886(0.0011)	220(0.4726)
RENBI	0.0875(0.0014)	0.0812(0.0009)	0.8990(0.0021)	0.3758(0.0008)	0.7923(0.0007)	243(0.3725)
CSI	0.0970(0.0017)	0.0759(0.0010)	0.9278(0.0014)	0.3315(0.0006)	0.7530(0.0006)	200(0.3718)
CRE	**0.0830(0.0011)**	**0.0835(0.0009)**	**0.9383(0.0011)**	**0.3034(0.0006)**	**0.8329(0.0005)**	**169(0.3507)**
Netflix	〈*r*〉	*P*	*AUC*	*I*	*H*	〈*k*〉
CF	0.1755(0.0004)	0.0235(0.0003)	0.8714(0.0021)	0.3106(0.0009)	0.6787(0.0010)	423(1.2803)
NBI	0.1661(0.0004)	0.0251(0.0003)	0.8858(0.0019)	0.2819(0.0008)	0.7299(0.0006)	398(1.0763)
HNBI	0.1554(0.0004)	0.0270(0.0004)	0.8860(0.0021)	0.2521(0.0005)	0.8414(0.0005)	339(0.8053)
RENBI	0.1220(0.0003)	**0.0364(0.0003)**	0.9131(0.0019)	0.2373(0.0005)	0.8952(0.0003)	295(0.5911)
CSI	0.1437(0.0003)	0.0310(0.0004)	0.9063(0.0016)	0.1937(0.0012)	0.9063(0.0003)	256(0.7554)
CRE	**0.1191(0.0003)**	0.0356(0.0004)	**0.9154(0.0017)**	**0.1629(0.0003)**	**0.9480(0.0002)**	**198(0.4003)**
Amazon	〈*r*〉	*P*	*AUC*	*I*	*H*	〈*k*〉
CF	0.1212(0.0010)	0.0156(0.0001)	0.8810(0.0017)	0.0927(0.0001)	0.8649(0.0008)	81(0.1938)
NBI	0.1170(0.0011)	0.0162(0.0001)	0.8844(0.0018)	0.0899(0.0001)	0.8619(0.0006)	82(0.1775)
HNBI	0.1169(0.0011)	0.0162(0.0002)	0.8843(0.0019)	0.0896(0.0001)	0.8653(0.0007)	81(0.1182)
RENBI	0.1103(0.0012)	0.0181(0.0002)	0.8848(0.0019)	**0.0861(0.0001)**	0.9245(0.0004)	68(0.1182)
CSI	0.1036(0.0011)	0.0190(0.0001)	0.8930(0.0018)	0.0880(0.0002)	0.9667(0.00007)	48(0.0479)
CRE	**0.1036(0.0011)**	**0.0190(0.0001)**	**0.8930(0.0018)**	0.0880(0.0002)	**0.9667(0.00007)**	**48(0.0479)**

**Table 2:** Performance comparison table. The optimal *α*’s of ranking score 〈*r*〉 for HNBI, RENBI, CRE are (-0.86, -0.76, -0.93) in Movielens, (-1, -0.81, -0.88) in Netflix and (-0.08, -0.53, 0) in Amazon, respectively. And other evaluation metrics—*P* for precision, AUC, *I* for intra-similarity, *H* for hamming distance, 〈*k*〉 for popularity— take the values corresponding to the optimal *α* of 〈*r*〉. The recommendation list *L* = 100, and the sampling number *n* in *AUC* is one million. All the values are obtained by averaging over ten independent runs with different data set divisions and numbers in brackets stand for the standard deviations.

Movielens	〈*r*〉	*P*	*AUC*	*I*	*H*	〈*k*〉
CF	0.1225(0.0020)	0.0443(0.0006)	0.8990(0.0020)	0.3336(0.0007)	0.4826(0.0013)	205(0.3754)
NBI	0.1143(0.0019)	0.0461(0.0006)	0.9093(0.0017)	0.3153(0.0006)	0.5209(0.0011)	199(0.3773)
HNBI	0.1075(0.0018)	0.0478(0.0006)	0.9144(0.0014)	0.3004(0.0007)	0.5946(0.0012)	189(0.3378)
RENBI	0.0875(0.0014)	0.0542(0.0006)	0.9349(0.0013)	0.2722(0.0004)	0.7301(0.0007)	156(0.2666)
CSI	0.0970(0.0017)	0.0512(0.0007)	0.9278(0.0014)	0.2829(0.0005)	0.6743(0.0006)	171(0.2479)
CRE	**0.0830(0.0011)**	**0.0551(0.0005)**	**0.9383(0.0011)**	**0.2511(0.0004)**	**0.7797(0.0007)**	**140(0.3226)**
Netflix	〈*r*〉	*P*	*AUC*	*I*	*H*	〈*k*〉
CF	0.1755(0.0005)	0.0186(0.0002)	0.8714(0.0022)	0.3034(0.0007)	0.6167(0.0010)	378(0.9545)
NBI	0.1661(0.0004)	0.0197(0.0002)	0.8859(0.0020)	0.2772(0.0006)	0.6727(0.0007)	358(0.8371)
HNBI	0.1554(0.0004)	0.0212(0.0002)	0.8860(0.0022)	0.2529(0.0005)	0.7893(0.0007)	313(0.6651)
RENBI	0.1220(0.0003)	**0.0275(0.0002)**	0.9131(0.0020)	0.2303(0.0003)	0.8667(0.0002)	265(0.3904)
CSI	0.1437(0.0003)	0.0236(0.0002)	0.9063(0.0016)	0.1998(0.0003)	0.8661(0.0003)	249(0.4804)
CRE	**0.1191(0.0003)**	0.0272(0.0002)	**0.9154(0.0017)**	**0.1659(0.0002)**	**0.9226(0.0002)**	**193(0.3100)**
Amazon	〈*r*〉	*P*	*AUC*	*I*	*H*	〈*k*〉
CF	0.1212(0.0011)	0.0109(0.0001)	0.8811(0.0018)	0.0730(0.0001)	0.8309(0.0006)	71(0.1037)
NBI	0.1170(0.0011)	0.0113(0.0001)	0.8844(0.0019)	0.0706(0.0001)	0.8287(0.0006)	72(0.1163)
HNBI	0.1169(0.0011)	0.0113(0.0001)	0.8843(0.0018)	0.0703(0.0001)	0.8323(0.0006)	71(0.1099)
RENBI	0.1103(0.0012)	0.0123(0.0001)	0.8848(0.0019)	**0.0669(0.0001)**	0.9010(0.0002)	60(0.0596)
CSI	0.1036(0.0011)	0.0128(0.0001)	0.8936(0.0018)	0.0685(0.0001)	0.9467(0.0001)	46(0.0530)
CRE	**0.1036(0.0011)**	**0.0128(0.0001)**	**0.8936(0.0018)**	0.0685(0.0001)	**0.9467(0.0001)**	**46(0.0530)**

More concretely, let’s analyze Table 1 at first. Evidently, CRE surpasses CF the most in all aspects, especially even with 〈*r*〉 reduced by more than 32%, *H* increased by more than 44% in Movielens, *P* increased by more than 51%, *I* increased by more than 47% and 〈*k*〉 reduced by more than 53% in Netflix, and in Amazon, besides, *P* increased by more than 23% and 〈*k*〉 reduced by more than 51%. CRE transcends NBI on six metrics, distinctively, with 〈*r*〉 reduced by more than 28%, *P* increased by more than 41% and *I* reduced by 42% in Netflix, *H* increased by more than 34% in Movielens and 〈*k*〉 reduced by more than 52% in Amazon. CRE is superior to HNBI on all six metrics, with 〈*r*〉 reduced by more than 23%, *P* increased by more than 31%, *I* reduced by 41% in Netflix, *H* increased by more than 21% in Movielens, and 〈*k*〉 reduced by more than 51% in Amazon. CRE is more excellent than RENBI in most cases. Prominently, herein, CRE overcomes RENBI with *I* reduced by more than 31% in Netflix, and 〈*k*〉 reduced by more than 42% in Amazon. At last, CRE stands on top of CSI, remarkably with 〈*r*〉 reduced by more than 17%, *P* increased by more than 15%, *I* reduced by more than 23% and 〈*k*〉 reduced by more than 23% in Netflix, *H* increased by more than 11% in Movielens.

In addition, from further examination in Table 2 with *L* = 100, CRE also has approximately similar performances corresponding to Table 1. Even though there exists differences in three benchmark data sets, we argue that CRE obviously outperforms the five mainstream baselines in diversity and personality, and meanwhile has excellent accuracy in most cases as well. Especially, in data set Amazon containing little redundancy because of diversity of goods for sale, CRE shows the optimal values as the same as CSI at *α* = 0, meaning that it degrades to CSI. In others words, from the definition of CRE, it is obvious that CSI is a special case of CRE, suggesting CRE a more capable and adaptive algorithm in various conditions of different datasets to approach the satisfied performances.

### Analysis

To better reveal the intrinsic nature that CRE outperforms benchmark methods, we compare the recommendation processes of all methods. Generally, CF makes recommendation reasonably based on similarity between users, but still ranks with the worst compared with CRE because it neglects the similarity between objects and users’ similarity redundancy. NBI distinctively performs better than CF but also shows severe shortage in contrast to CRE. It is due to the unidirectional defective (or apparent) similarity between objects and neglects similarity redundancy. HNBI and RENBI are the derivations of NBI. HNBI only penalizes the high degree of popular objects and RENBI only eliminates the similarity redundant. However, both of them are based on the unidirectional defective similarity between objects like NBI. To the opposite, CSI explicitly corrects the biased unidirectional similarity, but preserves the original adverse similarity redundancy. Meanwhile, it brings in secondary redundancy because of the accumulation of bidirectional similarities, which may lead to much worse redundancy.

These traditional similarity based algorithms indeed either contains the analogous drawback of similarity estimation, or hides with annoying similarity redundancy, which cause unsatisfied recommendation performance. Nevertheless, CRE simultaneously combines apparent similarity correction with redundant similarity elimination, even removes secondary redundant similarity brought by similarity correction procedure. It surely achieves the admirable improvements in accuracy, diversity and personality. More importantly, CRE intrinsically holds the merits of our proposed CSI, but modifies its defects. Moreover, CRE with tunable parameter can adjust to diverse similarity redundancies for the most suitable recommendations.

Besides, the lower computation complexity is another important factor when we design the recommendation algorithm. As we known, the time complexity of product of two *N* × *N* matrices is *O*(*N*^3^). To NBI and CSI without searching precess, they have the complexity of *O*(*N*^3^). However, even though necessary for searching for optimal value, compared with *N*, the searching cost is negligible. Accordingly, CRE, HNBI and RENBI still retain the complexity as *O*(*N*^3^), implying great improvement of performance but without increasing complexity.

## Discussions

We have investigated the similarity based recommendation algorithms (mainly involving with benchmark methods) and find the existence of two problem, that is, apparent similarity estimations due to only considering unidirectional mass diffusion and similarity redundancy caused by the correlations between objects’ attributes. Even the worse, in some benchmark methods, such as CF, NBI, HNBI, they both originally exist. Significantly, CSI bring in secondary similarity redundancy to make recommendation worse in some evaluation metrics, such as *I*, *H*, 〈*k*〉 in Movielens, although it correct the apparent similarity. After exploring biased unidirectional similarities from the collected objects to the uncollected ones and similarity redundancies derived from correlations between objects’ attributes, a corrected redundancy-eliminating model (i.e., CRE) is proposed. Herein, modeled with symmetrical mass diffusion, CRE believes stronger symmetric mass diffusion makes more precise similarity estimation. Additionally, CRE advisably eliminates unexpected original and secondary similarity redundancy caused by mass diffusion. Through experimental verifications on three benchmark datasets, CRE indeed achieves great and impressive improvement in accuracy, diversity and personality in comparison with other methods. Because of high effectiveness and low complexity, CRE can be applied in various kinds of recommendation systems, such as online news recommendation, online books recommendation, online movies recommendation, online music recommendation, and so on. Although obtaining great improvement, CRE still has weaknesses. For example, the lack of consideration on node degrees may to some extent impacts the recommendation performance. This will be further investigated in our future work.

## Data & metrics

### Data

Three real benchmark datasets, *Movielens* from http://www.grouplens.org/, *Netflix* from http://www.netflix.com/, and *Amazon* from http://www.amazon.com/ are introduced to demonstrate the effectiveness of our CRE index and freely downloaded from KONECT database. Three benchmark datasets are firstly realeased by GroupLens, Netflix and Amazon, which mainly used to testing recommendation alogorithms. They are gathered into the KONECT database built by Institute of Web Science and Technologies at the University of Koblenz-Landau (http://konect.uni-koblenz.de/networks/). The aim of the KONECT database is for public academic research. We guarantee that there is no conflict of interest. And, all people can freely download these datasets. *Movielens* and *Netflix* are well-known movie recommendation websites, and *Amazon* is a famous online shopping store. Ratings in such web sites are extracted to rank users’ preference to the objects with extent from 1 to 5 stars. We believe user likes the object if he/she rank the ratings ≥ 3, and then the rest dislike links will be abandoned. Consequently, we can gain the ultimate processed experimental datasets, detailed in the following Table 3.

**Table 3:** Summary on primary information of four datasets.

Data	Users	Objects	Links	Sparsity
Movielens	943	1682	1000000	6.3 × 10^−1^
Netflix	10000	6000	701947	1.17 × 10^−2^
Amazon	3604	4000	134679	9.24 × 10^−3^

For the sake of clear description of experiment, we denote all the possible user-object links as a whole link-set *E*^*A*^. Further, we divide the existed link-set *E* into training set *E*^*T*^ with 90% links of the total and probe set *E*^*P*^ with the remaining 10% links (*E*^*P*^ \ *E*^*T*^ = ∅). It is noticed that links in the probe set are considered as unknown information which is prohibited from taking in training phase. The links in the set *E*^*A*^ \ *E* represent all the unrealized user-object selections.

### Evaluation

For evaluating the recommendation performance, we focus on three categories of metrics: accuracy, diversity and personality (contrary to popularity) [14].

The accuracy is usually assessed by three metrics, including averaged ranking score, precision and AUC, which are described as follows:

1. Averaged ranking score (〈*r*〉): Better ranking score is smaller, meaning all the links in the probe set *E*^*P*^ are ranked ahead contrast to the links in the set *E*^*A*^ \ *E*^*T*^. If *u*_*j*_ purchases *o*_*i*_ in the *E*^*P*^ and the link gets the ranking position *p*_*ij*_ in his/her uncollected objects set *O*_*j*_ based on the recommendation score, we obtain the $rank_{ij}=\frac{p_{ij}}{\left|O_{j}\right|}$ as the ranking score of *o*_*i*_-*u*_*j*_ link *l*_*ij*_. Consequently, we compute the averaged ranking score 〈*r*〉 via all the links in *E*^*P*^ as follows:
  $$\langle r\rangle \begin{array}{c} =\frac{\sum_{l_{ij}\in E^{P}}rank_{ij}}{\left|E^{P}\right|} \end{array}$$ (3)
  Where |*O*_*j*_| and |*E*^*P*^| suggest the cardinality of sets.
2. Precision (*P*): If a user *u*_*j*_ has *N*_*j*_ recommended testing links, the precision *P*_*j*_(*L*) of him/her equals to $\frac{N_{j}}{L}$ with recommendation list length = *L*. Furthermore, the precision *P* of the whole system can be calculated through all users’ individual precisions as
  $$\begin{array}{c} P=\frac{1}{m}\sum_{j=1}^{m}P_{j}\left(L\right) \end{array}$$ (4)
3. Area Under ROC Curve (AUC): AUC is designed for the measurement that a recommender system can effectively discriminate the users’ appreciated objects from all other objects. There exists a convenient way to compute AUC, we can compare the probability that the users’ appreciated objects will be recommended with that of the uninterested objects. In *n* independent comparisons (each comparison means choosing an appreciated and a disliked object), if the appreciated object has *n*′ times higher score than the disliked and *n*′′ times equal, then
  $$\begin{array}{c} AUC=\frac{n^{\prime }+0.5n^{\prime \prime }}{n} \end{array}$$ (5)
  Evidently, if all appreciated objects are ranked higher score than the opposite objects, AUC = 1 which implies a perfect recommendation list. For a completely random recommendation list, AUC = 0.5. Therefore, the more AUC exceeds 0.5, the more excellent the ability of a recommendation algorithm to distinguish niche objects.

Referred to diversity, we usually consider intra-similarity and hamming distance, which are introduced as below:

1. Intra-similarity (*I*): A single user should be recommended with diverse objects [38] to avoid dullness and attract his/her interests. Otherwise such method would degrade user’s loyalty for receiving boring recommendation under the same topic. Thus, for a certain target user *u*_*l*_, we set the recommended objects for *u*_*l*_ as {*o*_1_, *o*_2_, …, *o*_*L*_}. By S*ϕ*ensen index [39], the similarity between *o*_*i*_ and *o*_*j*_ can be modeled as,
  $$\begin{array}{c} s_{ij}^{o}=\frac{1}{\sqrt{k\left(o_{i}\right)k\left(o_{j}\right)}}\sum_{l=1}^{m}a_{il}a_{jl} \end{array}$$ (6)
  *k*(*o*_*i*_) is item *i*’s degree. In addition, we can define the intra-similarity of *u*_*l*_’s recommendation list as,
  $$\begin{array}{c} I_{l}=\frac{1}{L(L-1)}\sum_{i\neq j}s_{ij}^{n} \end{array}$$ (7)
  The whole system’s intra-similarity is thus calculated as,
  $$\begin{array}{c} I=\frac{1}{m}\sum_{l=1}^{m}I_{l} \end{array}$$ (8)
2. Hamming distance (*H*): Another perspective to see the diversity of recommendations is the intra-diversity, which is quantified via the Hamming distance and the essence of personalized recommendations. Assumed the recommendation list length = *L* (i.e., the number of objects recommended to each user), if the overlapped number of objects in *u*_*i*_ and *u*_*j*_’s recommendation lists is *Q*, their recommendation lists’ Hamming distance is described as,
  $$\begin{array}{c} H_{ij}=1-Q/L \end{array}$$ (9)
  In a word, a more personalized recommendation list should be qualified with larger Hamming distances contrast to other lists. Accordingly, we can further measure the diversity of recommendations through Hamming distance as
  $$\begin{array}{c} H=\frac{1}{m(m-1)}\sum_{i\neq j}H_{ij} \end{array}$$ (10)
  averaged over all the user-user pairs. Note that, *H* only takes into account the diversity among users.

The popularity is estimated by average degree over recommended objects to represent personality:

1. Average degree (〈*k*〉): *o*_*ij*_ is the j*th* recommended item as to user *i*. *k*(*o*_*ij*_) denotes item *o*_*ij*_’s degree. We can leverage the average degree of all recommended items for all users to compute the popularity as below,
  $$\begin{array}{c} <k>=\frac{1}{mL}\sum_{i=1}^{m}\sum_{j=1}^{L}k\left(o_{ij}\right) \end{array}$$ (11)

### 2.3 Benchmark methods

Five mainstream indices, cooperative filtering (CF), network based inference (NBI), heterogenous initial resource distribution NBI (HNBI), redundancy elimination NBI (RENBI), corrected similarity index (CSI) are introduced for comparison with our CRE, listed as below:

1. Collaborative filtering (CF) [27]: Collaborative filtering is designed to compute similarity between users or objects. We define the cosine similarity between two users *u*_*i*_ and *u*_*j*_ as:
  $$\begin{array}{c} s_{ij}=\frac{1}{\sqrt{k\left(u_{i}\right)k\left(u_{j}\right)}}\sum_{l=1}^{n}a_{li}a_{lj} \end{array}$$ (12)
  For arbitrary user-object pair *u*_*i*_ − *o*_*j*_, when *u*_*i*_ has not yet collected *o*_*j*_ (i.e., *a*_*ji*_ = 0), the predicted score, *v*_*ij*_ (to what extent *u*_*i*_ likes *o*_*j*_), is calculated as
  $$\begin{array}{c} v_{ij}=\frac{\sum_{l=1,l\neq i}^{m}s_{li}a_{jl}}{\sum_{l=1,l\neq i}^{m}s_{li}} \end{array}$$ (13)
  To any user *u*_*i*_, we sort all the nonzero *v*_*ij*_ with *a*_*ji*_ = 0 in a descending order, and recommend those objects in the top-*L*.
2. Network Based Inference (NBI) [29]: NBI based on network structure computes the S*ϕ*ensen index. For a general user-object network, we can give the similarity weight between *o*_*i*_ and *o*_*j*_ as:
  $$\begin{array}{c} w_{ij}^{NBI}=\frac{1}{k(o_{j})}\sum_{l=1}^{m}\frac{a_{il}a_{jl}}{k(u_{l})} \end{array}$$ (14)
  where $w_{ij}^{NBI}$ comes from similarity weight matrix *W*^*NBI*^, and $k\left(o_{j}\right)=\sum_{i=1}^{m}a_{ji}$ and $k\left(u_{l}\right)=\sum_{i=1}^{n}a_{il}$ respectively denote the degrees of object *o*_*j*_ and user *u*_*l*_. Accordingly, we can obtain the recommendation list of user *u*_*l*_ as $f_{l}^{\prime }=W^{NBI}f_{l}$, with *f*_*l*_ = *a*_*li*_ representing the historical record of *u*_*l*_.
3. Heterogeneous NBI (HNBI) [30]: HNBI based on NBI takes heterogenous initial resource configuration into account with weight $w_{ij}^{HNBI}=k\left(o_{j}\right)w_{ij}^{\alpha }$. *w*_*ij*_ is from Eq (14) and $W^{HNBI}=\left\{w_{ij}^{HNBI}\right\}$. With purchase history *f*_*j*_ of *u*_*j*_, the probable recommendation list of *u*_*j*_ is $f_{j}^{\prime }=W^{HNBI}f_{j}$.
4. Redundancy-Eliminating NBI (RENBI) [31]: RENBI based on NBI further consider to eliminate the similarity redundancy. Say the similarity matrix of NBI as *W*, the similarity matrix of RENBI is modeled as *W*^*RENBI*^ = *W* + *αW*^2^ and the future recommendation list of *u*_*j*_ is acquired as $f_{j}^{\prime }=W^{RENBI}f_{j}$.
5. Corrected Similarity Index (CSI) [33]: CSI based on NBI further corrects unidirectional similarity. Given similarity matrix *W* = {*w*_*ij*_} of NBI, the forward similarity proportion is:
  $$\begin{array}{c} r_{ij}^{F}=\frac{w_{ij}}{\sum_{i=1}^{n}w_{ij}}=w_{ij} \end{array}$$ (15)
  and the back similarity proportion is:
  $$\begin{array}{c} r_{ji}^{B}=\frac{w_{ji}}{\sum_{j=1}^{n}w_{ji}}=r_{ji}, \end{array}$$ (16)
  eventually getting the CSI similarity *S*^*CSI*^ = {*s*_*ij*_} as:
  $$\begin{array}{c} s_{ij}=\sqrt{r_{ij}^{F}\times r_{ji}^{B}}. \end{array}$$ (17)
  Therefore, the recommendation list of *u*_*j*_ is $f_{j}^{\prime }=S^{CSI}f_{j}$.
